# Suppressive Effect of Juzen-Taiho-To on Lung Metastasis of B16 Melanoma Cells *In Vivo*

**DOI:** 10.1093/ecam/nen081

**Published:** 2011-06-05

**Authors:** Takako Matsuda, Katsuhiko Maekawa, Kazuhito Asano, Tadashi Hisamitsu

**Affiliations:** Department of Physiology, School of Medicine, Showa University, Tokyo, Japan

## Abstract

Juzen-Taiho-To (JTT) is well known to be one of Kampo (Japanese herbal) medicine consisted of 10 component herbs and used for the supplemental therapy of cancer patients with remarkably success. However, the precise mechanisms by which JTT could favorably modify the clinical conditions of cancer patients are not well defined. The present study, therefore, was undertaken to examine the possible mechanisms of JTT on prevention of cancer metastasis using experimental mouse model. JTT was well mixed with rodent chow at concentrations of either 0.2 or 1.0%, and administered orally *ad libitum*, which was started 1 week before tumor cell injection and continue throughout the experiment. Oral administration of JTT at concentration 0.2 and 1.0% into C57BL/6 male mice significantly inhibited tumor metastasis in lungs, which was induced by the intravenous injection of 2 × 10^5^ B16 melanoma cell. JTT at a concentration of 1.0% also significantly suppressed lung metastasis of B16 melanoma cell from hind footpad in C57BL/6 mice. In the second part of experiments, the influence of the depression of natural killer (NK) cell, natural killer T (NKT) cell and several types of cytokines on JTT-mediated inhibition of tumor cell metastasis. Intraperitoneal injection of anti asialo-GM1 antibody against NK cells and anti NK-1.1 monoclonal antibody (mAb) to NKT cells abrogated the inhibitory action of JTT on lung metastasis of B16 melanoma cells. Although intraperitoneal administration of anti-IFN-*γ* mAb scarcely affected the inhibitory action of JTT on tumor cell metastasis, injection of amrinone, which used for IL-12 suppression, significantly decreased the ability of JTT to prevent tumor cell metastasis. These results strongly suggest that oral administration of JTT caused increase in the production of IL-12, which is responsible for the activation of both NK cell and NKT cell, in the lungs and results in inhibition of B16 melanoma cell metastasis in the lungs.

Digestive cancer patients generally undergo surgical therapy, chemotherapy, radiotherapy or a combination of these treatments. While the effects of these treatments are significant, it is a fact that most patients suffer from side-effects, such as high fever, general fatigue, loss of appetite, pancytopenia and many kinds of infections.

In Japan and China, herbal medicine is used as a supplemental therapy for many kinds of chronic diseases such as loss of appetite, anemia and chilliness of the arms and legs with remarkable success [1, 2]. Recent reports clearly showed that when herbal medicines are used for cancer treatment, many patients experience fewer or diminished side-effects induced by western medicine, such as chemotherapy and radiotherapy, and the survival period is longer [3, 4]. It is also reported that herbal medicine can prevent the progression of colon carcinoma, gastric and breast cancer as well as the prevention of these cancer metastasis in liver, lung or bone [3, 4]. Moreover, hepatocellular carcinoma has been shown to become smaller without severe side-effects [5]. Although these reports strongly suggest that herbal medicine will be a good alternative for the treatment of several types of cancer, the mechanisms by which herbal medicine could improve clinical status, including cancer metastasis.

The immune system of vertebrates is composed of two major arms: innate immunity and adoptive immunity. Innate immunity provides a swift response to agents prior to the initiation of adoptive immune responses. The innate response is almost immediate and is based on cells and soluble mediators that recognize common molecular patterns on pathogenic bacteria and transformed cells. Cells involved in the innate immune responses include several types of cells, such as macrophages, dendritic cells, natural killer (NK) cells and natural killer T (NKT) cells. In these phagocytic cells, NK and NKT cells play essential roles in tumor immunology: NK cells have killer activating receptors and cause lysis of target cells using specific enzymes, perforin and granzymes [6]. NKT cells, which are cytotoxic cells that have characteristics of both NK cells and T cells, play a role in searching for tumor cells, so called immune surveillance, and in preventing metastasis [7]. From these reports, it is reasonable to speculate that oral administration of herbal medicine in cancer patients enhance the activity of NK and NKT cells and results in both prevention of cancer metastasis and favorable modification of clinical conditions of the patients. However, there is no evidence showing that herbal medicine can enhance the function of both NK and NKT cells *in vivo*. The present study, therefore, was undertaken to examine the influence of herbal medicine on NK and NKT cells using Juzen-Taiho-To (JTT) and experimental cancer metastatic model in mice.

## 1. Methods

### 1.1. Mice

Specific pathogen-free C57BL/6 male mice, 6-week old, and ICR nude female mice (nu/nu; 5 weeks of age) were purchased from Japan Bio-Supply Center (Tokyo, Japan). The animals were maintained at 25 ± 2°C, humidity 50 ± 2%, and a light and dark cycle of 12 h in our animal facilities. The mice were randomly divided into groups of five mice and fed chow containing JTT (0.2% or 1%) or regular diet (control). This study was approved by the Ethics Committee of Showa University for Animal Experiments.

### 1.2. Reagents and Antibodies

JTT was provided by Tumura Co. Ltd. (Tokyo, Japan) as a preservative free pure powder. JTT is composed of 10 herbs: *Astragali Radix, Cinamomi Cortex, Rehmanniae Radix, Paeoniae Radix, Cnidii Rhizoma, Angelicae radix, Ginseng radix, Hoelen, Glycyrrhizae Radix and Atractylodis Lanceae Rhizoma*. Amrinone (Sigma Chemicals, St Louis, MO, USA) was dissolved in 0.5 N lactic acid at a concentration of 5%, diluted with phosphate buffered saline to the total volume of 10 ml, and then sterilized by passing through a 0.2-mm filter [8]. Anti-asialo-GM1 was obtained from Wako Pure Chemicals (Osaka, Japan). Monoclonal antibody (mAb) against IFN-*γ* was purified by ammonium sulfate precipitation [9] from ascites collected from ICR- nude mice inoculated with rat hybridoma R4-6A2 (kindly donated by Dr Y. Kudo, Tohoku University, Sendai, Japan). The mAb against NK1.1 was kindly donated from Dr N. Watanabe (Jikei University, Tokyo, Japan).

### 1.3. Preparation of Diet Containing JTT

Pure powder of JTT was well mixed with normal powder diet (MF) for maintaining rats and mice (Oriental Kobo Kogyou Co. Ltd., Tokyo, Japan) at concentrations of either 0.2 or 1.0%. The concentration of JTT in the diet (0.2%) is equivalent to the clinical dose (7.5 g/day/50 kg) [10].

### 1.4. Tumor Cells

B16 melanoma cells were purchased from Dai-Nippon Pharmaceutical Co. Ltd. (Osaka, Japan) and maintained with RPMI-1640 medium supplemented with 10% heat inactivated fetal calf serum (Flow Laboratories, North Ride, Australia).

### 1.5. Assay for Tumor Cell Metastasis

In the first experiments, B16 cells (2 × 10^5^) were injected subcutaneously into the right hind sole in a volume of 0.l ml. After tumor growth reached *∼*1 cm (*∼*14 days after cell injection), the tumor was removed under a dissecting microscope. These mice were then maintained for further 21 days and the black dots, showing tumor colony formation, on the lung surface were counted under a dissecting microscope [11, 12]. In the second experiments, B16 cells (2 × 10^5^) were injected intravenously into recipient mice in a volume of 0.1 ml. After 14 days, mice were killed under ether anesthesia and the number of tumor colonies on the lung surface was counted in a similar manner. In these two experiments, mice were given food containing JTT and tap water *ad libitum* for 2 or 3 weeks starting 7 days before tumor cell injection.

### 1.6. Assay for Cell Cytotoxicity of JTT

B16 melanoma cells (1 × 10^4^ cells/ml) were introduced in triplicate into 24-well culture plates that contained various concentrations of JTT. The number of cells was counted with trypan blue 24 and 48 h after culture.

### 1.7. Depression of NK Cell and NKT Cell

To depress NK cells and NKT cells, anti-NK1.1 mAb was injected intraperitoneally into mice at a single dose of 10 mg/kg on Days −2, 0, 2 and 7 relative to tumor cell injection [13]. In a case of NK cell depression, anti-asialo-GM1 antibody was injected intraperitoneally into mice at a dose of 200 mg/mouse 1 day after tumor cell injection [14].

### 1.8. Depression of IFN-*γ* and IL-12

To depress IFN-*γ*, anti-IFN-*γ* mAb was injected into mice on Days 3, 0, 3, 7, 9, 11, 14 relative to tumor cell injection at a single dose of 50.0 mg/day. To suppress IL-12, amrinone was given intraperitoneally every day from Day −1 to 14 at a single dose of 100 mg/day [8] in a volume of 0.4 ml.

### 1.9. Statistical Analysis

The statistical significance between control and experimental groups was analyzed with analysis of variance (ANOVA) followed by Fisher's PLSD test. *P* < .05 was considered significant.

## 2. Results

### 2.1. Suppression of B16 Melanoma Cell Metastasis by JTT

**Figure 1:**
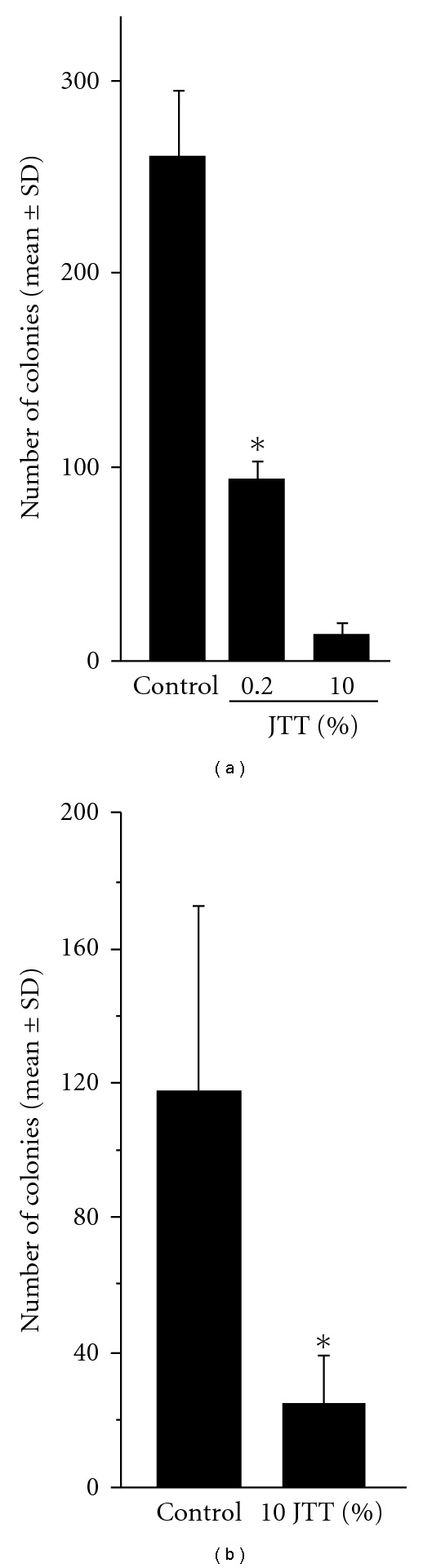
Influence of JTT on B16 melanoma cell metastasis in mice. C57BL/6 mice were orally administered JTT, which was started 1 week before injection of 2 × 10^5^ melanoma cells and killed 3 or 5 weeks later to count tumor cell colonies in the lungs. (a) Number of colonies in the lungs 2 weeks after intravenous injection of cells. (b) Number of tumor colonies in the lungs 5 weeks after subcutaneous injection of cells. ^+^ *P* < .05.

This experiment was undertaken to examine the influence of oral administration of JTT on tumor cell metastasis using different two types of experimental models. Mice pretreated with either 0.2% or 1.0% JTT for 7 days were injected intravenously with 2 × 10^5^ B16 cells and were killed 14 days later to count the number of tumor cell colonies in the lungs. As shown in Figure 1(a), oral administration of 0.2% JTT could cause significant suppression of B16 melanoma cell metastasis. Furthermore, this suppressive activity of JTT on tumor metastasis was further strengthened when mice were pretreated with 1.0% JTT: the number of tumor cell colonies observed in mice treated with 1.0% JTT was much lower than that in 0.2% JTT-treated mice. We then examined whether oral administration of JTT could also prevent spontaneous tumor cell metastasis as in the case of intravenous administration of tumor cells. As shown in Figure 1(b), administration of 1.0% JTT into mice could prevent spontaneous B16 melanoma cell metastasis from right hind footpad to lung surface.

### 2.2. Influence of JTT on Tumor Cell Growth *in vitro*

The present study was designed to determine whether JTT exerts cytotoxic effects on B16 melanoma cells and results in the prevention of tumor cell metastasis. As shown in Table 1, JTT could not suppress B16 melanoma cell growth even when cells were cultured in the presence of 25% JTT: the number of cells in experimental cultures observed at 48 h is almost equal to that observed in control cultures.

### 2.3. Effect of NK Cell and NK-1.1 Cell Depression on Tumor Cell Metastasis in JTT-Treated Mice

**Figure 2:**
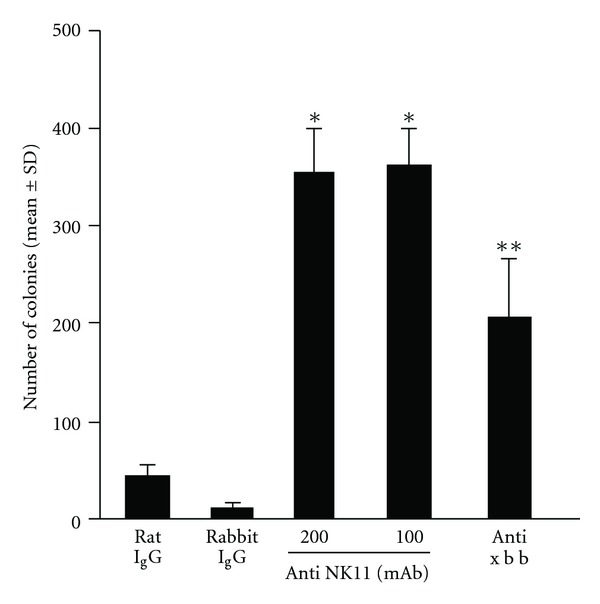
Influence of injection of antibodies against natural killer or natural killer T cells on B16 melanoma cell metastasis in mice treated with JTT. C57BL mice were orally administered with 1.0% JTT for 3 weeks, which was started 1 week before 2 × 10^5^ melanoma cell injection. Anti-NK1.1 monoclonal antibody or anti-asialo-GM1 antibody was injected intraperitoneally and number of tumor cell colonies was counted 2 weeks later. ^+^ *P* < .05, ^++^ *P* < .01.

This experiment was carried out to examine the influence of NK cell and NK-1.1 cell depression on tumor cell metastasis. To do this, recipient mice were given either anti-asialo-GM1 or anti NK-1.1 mAb, and challenged intravenously with B16 melanoma cells. After 14 days, the influence of cell depletion was assessed by counting the number of tumor cell colonies on lung surface. As shown in Figure 2, injection of anti asialo-GM into mice abrogated inhibitory action of JTT on tumor cell metastasis: the number of colonies in mice treated with antibody is significantly higher than that in control mice. Injection of anti NK-1.1 mAb also eliminated the ability of JTT to prevent tumor cell metastasis and higher number of colonies was observed in mAb-treated mice as compared with control.

### 2.4. Tumor Cell Metastasis in Cytokine-Depressed JTT-Treated Mice

**Figure 3:**
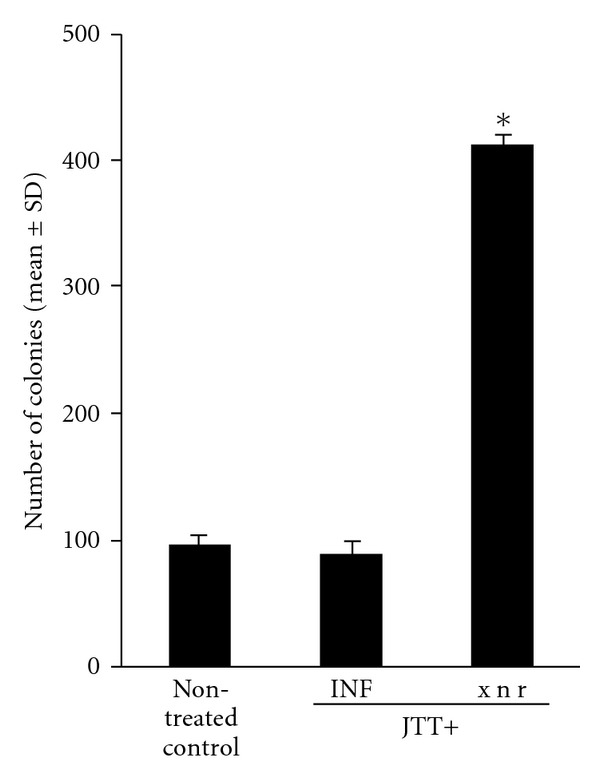
Influence of anti-IFN-*γ* monoclonal antibody or amrinone injection on B16 melanoma cell metastasis in mice treated with JTT. C57BL mice were orally administered with 1.0% JTT for 3 weeks, which was started 1 week before 2 × 10^5^ melanoma cell injection. Anti-IFN-*γ* monoclonal antibody or amrinone was injected intraperitoneally and number of tumor cell colonies was counted 2 weeks later. ^+^ *P* < .05.

This study was designed to examine the influence of cytokine depression on the prevention of tuxmor cell metastasis induced by 1.0% JTT administration. Mice were treated with either anti IFN-*γ* monoclonal antibody or amrinone and challenged with 2 × 10^5^ tumor cells. After 14 days, mice were sacrificed and the number of tumor cell colonies in the lung surface was counted. As shown in Figure 3, injection of anti-IFN-*γ* mAb scarcely affected the suppressive activity of JTT on tumor cell metastasis and the number of tumor cell colonies in JTT-treated, mAb-injected mice was similar to that observed in JTT-treated, non-mAb-injected mice. On the other hand, treatment of mice with amrinone caused significant suppression of the ability of JTT to prevent tumor cell metastasis and many metastatic nodules of the lung were observed in JTT-treated, amrinone-injected mice compared with JTT-treated, non-injected mice.

## 3. Discussion

Herbal medicine is used frequently as a supplemental therapy for many kinds of chronic diseases with remarkably success [1]. In cases of treatment for cancer, herbal medicine is reported to be able to prevent the progression of colon carcinoma, gastric and breast cancer as well as the prevention of the cancer metastasis to the liver, lung or bone [15]. However, the mechanisms by which herbal medicine could improve clinical status of cancer patients, including cancer metastasis. The present study, therefore, was undertaken to examine the possible mechanisms of herbal medicine on the prevention of cancer metastasis through the choice of JTT and B16 melanoma cell/mouse system *in vivo*. We regard JTT has an ability to prevent cancer metastasis by BRM (biological response modifierthe).

The present results showed that oral administration of JTT inhibited B16 melanoma cell colony formation on the lung surface, when the recipient mice were given tumor cells intravenously (Figure 1(a)). JTT also suppressed spontaneous B16 tumor cell metastasis from hind footpad to the lung surface (Figure 1(b)). The prevention of tumor cell growth and metastasis is well accepted to be through diverse mechanisms, including tumor cell death, apoptosis and immune-mediated cancer regression. Our results revealed the absence of cytotoxic effects of JTT on B16 melanoma cells (Table 1) suggesting that immune-mediated mechanisms are responsible for the prevention of tumor cell colony formation on the lung surface. The immune effector responses against tumor cells involve activity by several cellular constituents: (i) T cells carry out immunologic surveillance, then proliferates and destroys tumor cells after recognizing tumor-associated antigens in combination with major histocompatibility complex (MHC) molecules. (ii) Dendritic cells are important antigen presenting cells that can present antigen to both helper and cytotoxic T cells and are able to stimulate a naïve T cell response. (iii) NK and NKT cells are another populations of effector cells with tumoricidal activity. In contrast to cytotoxic T cells, NK cells can kill tumor cells in a non-MHC-dependent fashion. We, therefore, examined the final effector cells that cause tumor cell killing in JTT-treated mice. The present results proved that injection of NK-1.1 mAb and anti asialo-GM1 antibody eliminate the suppressive activity of JTT on tumor cell metastasis. Anti NK-1.1 mAb is reported to be the effective means of the depleting NK and NKT cells [16]. On the other hand, injection of anti-asialo-GM1 antibody into mice only eliminate NK cells from a variety of mouse strains [16]. Taken together, the present results may suggest that NK cells play essential roles in prevention of tumor cells metastasis in the lung.

Several cytokines have been shown to affect NK cell proliferation and cytolytic activity. Of these, IFN-*γ*, produced by activated T cells and NK cells, in conjunction with other cytokines such as IL-12, is thought to enhance the cytolytic activity of NK cells to attach and kill tumor cells [17–19], indicating that oral administration of JTT into mice increases the levels of cytokines, including IFN-*γ* and IL-12 in the lung tissues and results in prevention of tumor cell colony formation. On the other hand, the present results also open the question that whether IFN-*γ* or IL-12 is important for the development of the ability to prevent tumor metastasis observed in JTT-treated mice. The present results indicate that administration of neutralizing anti-IFN-*γ* mAb could not abrogate the suppressive activity of JTT on tumor cell metastasis. However, administration of amrinone, which cause specific suppression of IL-12 production [20], caused complete elimination of the ability of JTT to prevent tumor metastasis in the lung. These results strongly suggest that IL-12 is the main mediator in the development of inhibitory action on tumor metastasis observed in mice treated with JTT.

Although the present results indicated that JTT exerts the protective effects on tumor cell metastasis through enhancement of IL-12 production and NK cell activation, the component of JTT which shows immuno-modulatory effects, is not defined. There is evidence that oral administration of extracts from *A. radix*, a component of JTT, could enhance the ability of cells to produce several types of cytokines, which increase NK and NKT cell activity [21, 22]. It is also observed that *A. radix* directly activate NK cells to kill tumor cells *in vitro* [22–24], suggesting that *A. radix* is the most important component of JTT to prevent tumor cell metastasis. Further experiments are required to delineate the component, showing the suppressive activity of tumor cell metastasis *in vivo*.

The conclusions re-stated are as follows: (i) JTT could prevent tumor cell metastasis through the enhancement of NK cell activity, and (ii) This activity of JTT may be owing, in part, to its ability to increase in IL-12 production.

## Figures and Tables

**Table 1:** Influence of JTT on B16 melanoma cell growth *in vitro*.

Concentration of JTT (%)	No. of cells (mean ± SD × 10^4^ cells/ml)	
	24 h	48 h
0 (control)	3.5 ± 0.5	33.0 ± 4.0
10	4.0 ± 0.5	34.5 ± 8.3
25	4.0 ± 1.0	32.5 ± 9.3

B16 melanoma cells (1 × 10^4^ cells/ml) were cultured in the presence of either 0, 10 or 25% JTT. Viable cells were counted with haemocytometer in the presence of trypan blue.
